# The elevated visceral adiposity index increases the risk of hyperuricemia in Chinese hypertensive patients: A cross-sectional study

**DOI:** 10.3389/fendo.2022.1038971

**Published:** 2022-12-15

**Authors:** XiaoLi Song, Hui Liu, Jian Zhu, Wei Zhou, Tao Wang, Chao Yu, Lingjuan Zhu, Xiaoshu Cheng, Huihui Bao

**Affiliations:** ^1^ Department of Cardiovascular Medicine, The Second Affifiliated Hospital of Nanchang University, Nanchang, Jiangxi, China; ^2^ Central Hospital of Huanggang, Huanggang, Hubei, China; ^3^ Jiangxi Provincial Cardiovascular Disease Clinical Medical Research Center, Nanchang, Jiangxi, China; ^4^ Qiu Kou Town Central Health Center, Wuyuan, Jiangxi, China; ^5^ Center for Prevention and Treatment of Cardiovascular Diseases, The Second Affiliated Hospital of Nanchang University, Nanchang, Jiangxi, China

**Keywords:** visceral adiposity index, uric acid, hyperuricemia, hypertension, obesity

## Abstract

**Background:**

Uncertainty still remained about the relationship between visceral adiposity index (VAI) and hyperuricemia. The aim of this study was to investigate whether VAI was an independent risk factor for hyperuricemia in hypertensive Chinese patients.

**Methods:**

A cross-sectional study including 13176 hypertensive participants (6478 males) recruited from Wuyuan County, Jiangxi province, was conducted. All patients received anthropometric measurements, completed questionnaires and provided blood samples for biochemical testing. VAI was calculated by waist circumference, BMI, triglyceride and high-density lipoprotein cholesterol. Hyperuricemia was defined as serum uric acid ≥ 7 mg/dL in men and ≥ 6 mg/dL in women.

**Results:**

Overall, the average level of uric acid was 7.8 ± 2.0 mg/dL in males and 6.34 ± 1.78 in females and prevalence of hyperuricemia was 61.4% and 51.30%, respectively. In multivariate logistic regression analysis, the risk of hyperuricemia increased 1.77 times and 1.88 times with the increase of ln VAI in males (OR:1.77, 95% CI: 1.62, 1.94) and females (OR:1.88, 95% CI: 1.73, 2.04). For males, compared to quartile 1, the risk of hyperuricemia in the second, third and the forth quartile of visceral adiposity index were 1.34 (95% CI: 1.14, 1.57),1.82(95% CI: 1.54, 2.14) and 2.97 (95% CI: 2.48, 3.57). For females, compared to quartile 1, the risk of hyperuricemia in the second, third and the forth quartile of visceral adiposity index were 1.48 (95% CI: 1.28, 1.72), 1.99 (95% CI: 1.71, 2.32) and 2.92 (95% CI: 2.50, 3.42).

**Conclusions:**

This study found that VAI was an independent risk factor for hyperuricemia among hypertensive patients, which may provide some strategies for reducing the level of uric acid.

## Introduction

The improvement of living standards, changes in diet and living habits have gradually increased the incidence of hyperuricemia (1). Hyperuricemia, a critical public health issue, is not only a direct cause of gout, but also a risk factor for many cardiovascular diseases and their risk factors. Accumulated studies have demonstrate that high uric acid was associated with hypertension (2), heart failure (3, 4), diabetes (5), kidney disease (6), stroke (7) and coronary heart disease (8, 9). Moreover, increased uric acid was also an independent predictor of all-cause (10) and cardiovascular death (11). Consequently, it is critical to identify hyperuricemia timely and take measures to prevent disease in clinical practice.

Obesity is regarded as a common risk factor for hyperuricemia. Previous studies reported that some traditional obesity index, such as body mass index (BMI) (12), waist circumference (13), waist height ratio (WHtR) (14) and neck circumference (15) had a impact on serum uric acid metabolism. However, they does not have enough ability to distinguish between muscle and fat accumulation (16), as well as visceral fat and subcutaneous fat (17). In contrast to some traditional adiposity indices, such as body mass index (BMI), waist circumference(WC), in which VAI can take into account the effects of sex, triglycerides, and HDL cholesterol levels on body fat distribution and function, VAI has greatly improved the accuracy of visceral fat and its functional assessment, and is currently a valid index for evaluating metabolic diseases (18). Xiaolin Huang et al. demonstrated that VAI increased the risk of hyperuricemia among middle-aged and elderly Chinese adults (19). Another study consisting of 7632 adult subjects from the China Health and Nutrition Survey 2009 suggested that VAI was closely related to hyperuricemia (20). It has also been confirmed that VAI can predict the risk of hypertension (21). However, there are limited reports to explore the relationship between VAI and hyperuricemia in a population prone to adverse events such as hypertension, and we think it is necessary to clarify the exact association between VAI and hyperuricemia in hypertensive patients.

## Methods

### Study design and participants

This study was a sub-study of the China H-type Hypertension Registry Study (registration number: CHiCTR1800017274) carried out in Wuyuan County, Jiangxi Province. It was a real world and observational study with the aim of setting up a national H-type hypertension cohort study, investigating the incidence and treatment rate of H-type hypertension in China and evaluating the related risk factors. Those who met the following criteria were considered as qualified: 1) Aged 18 years or older; 2) Diagnosed with hypertension which defined as Systolic blood pressure (SBP)≥140mmHg and/or diastolic blood pressure (DBP) ≥90mmHg; or self-reported history of hypertension; or taking anti-hypertensive drugs); 3) Signing informed consent. From March 2018 to August 2018, our team recruited 14,268 participants altogether in Wuyuan County, Shangrao City, Jiangxi Province, China. Since VAI was calculated on triglycerides and HDL, we excluded patients taking blood lipid-lowering drugs (n=506) in order to avoid the influence of blood lipids itself. In addition, individuals with data missing (n=12) were excluded. 13, 176 participants included in final data analysis (**Supplementary Figure 1**).

### Data collection and measurement

At baseline, participants described the the information about sociodemographic characteristics (marital status, education, occupation), their living habits (smoking, drinking, physical exercise), medical history (hypertension, diabetes, chronic kidney disease, etc.) and medication through questionnaires. Weight, Height, circumference (WC) and hip circumference (HC) were measured by trained staff. Body mass index (BMI) was calculated by dividing body weight (kg) by the square of height (m). Blood pressure measurements for all patients would be performed three times with an electronic sphygmomanometer (Omron; Dalian, China) after 10 minutes of rest. The blood pressure in this study was the average of three blood pressure values. All individuals were informed to keep an empty stomach for more than 10 hours in order to collect their blood samples in the next morning and store them in the Biaojia Biotechnology Laboratory (Shenzhen). Biochemical indicators, including fasting blood glucose (FBG), total cholesterol (TC), triglycerides (TG), low-density lipoprotein cholesterol (LDL-C), high-density lipoprotein cholesterol (HDL-C), serum uric acid (SUA), homocysteine (Hcy) and creatinine were detected in the core laboratory of national kidney disease clinical research center (Guangzhou, China). The newly developed Chronic Kidney Disease-Epidemiology Collaboration equation was used to estimate glomerular filtration rate (22).

### Definitions

Hyperuricemia was defined as uric acid ≥ 7 mg/dL in men and ≥ 6 mg/ml in women (23). VAI was calculated according to the following formula (24):


VAI (males)=[WC/(39.68+1.89×BMI)]×(TG/0.81)×(1.52/HDL−C

VAI (females)=[WC/(36.58+1.89×BMI)]×(TG/1.03)×(1.31/HDL−C

### Statistical analysis

All data analysis used R software version 4.0.3 (https://www.R-project.org) and Empower (R) version 3.0 (www.empowerstats.com). A two-sided P < 0.05 was defined as significant differences. In the current study, continuous variables were showed in mean ± standard deviation (SD), while categorical variables were presented as percentage. T-test was used to compare continuous variables and Chi-square for categorical variables. The association between visceral adiposity index and hyperuricemia was assessed by logistic regression model. In detail, the current study constructed three models: unadjusted, partially adjusted (age), and fully adjusted (age, smoking, drinking, SBP, DBP, FBG, TC, LDL-C, eGFR, Hcy, anti-hypertensive drugs, glucose-lowering drugs). Generalized additive model (GAM) and smooth curve fitting (penalized spline method) were preformed to characterize the shape of the association between VAI and hyperuricemia clearly. Due to gender differences in serum uric acid levels, multiple regression and subgroup analysis were performed separately in men and women.

## Results

### Characteristics of participants

**Table 1** listed the baseline characteristics of males according to quartile of VAI. In general, the mean age was 63.79 ± 9.84 years old, and and the prevalence of hyperuricemia was 61.49%. The prevalence of hyperuricemia in first, second, third and fourth quartile was 49.88%, 57.75%, 64.48% and 73.83%, respectively. Patients with higher VAI values seemed to be younger, have higher level of WC, BMI, DBP, FBG, TC, TG, LDL, Hcy, and eGFR, but lower level of SBP, HDL-C. No difference was detected in history of CKD and number of people taking antihypertensive drugs. **Table 2** listed the baseline characteristics of females according to quartile of VAI. The mean age was 63.78 ± 8.99 years old, and and the prevalence of hyperuricemia was 51.30%. The prevalence of hyperuricemia in first, second, third and fourth quartile was 36.91%, 48.59%%, 55.56% and 64.14%, respectively. Significant differences were observed in age, drinking, WC, BMI, DBP, FPG, TC, TG, HDL-C, LDL-C and eGFR (all P values < 0.05). No difference was detected in the status of smoking, SBP, history of CKD and stroke.

**Table 1:** Baseline characteristics of study participants across visceral adiposity index (VAI) quartiles in males.

	Total participants	Quartile 1 (< 0.7)	Quartile 2 (0.7-1.1)	Quartile 3 (1.1-2.0)	Quartile 4 (≥ 2.0)	*P-*value
Characteristics	(n = 6478)	n = 1620	n = 1619	n = 1619	n = 1620
Age (years)	63.79 ± 9.84	67.20 ± 8.67	65.84 ± 9.32	62.93 ± 9.59	59.22 ± 9.82	<0.001
Smoking (n, %)	3165 (48.87%)	828 (51.11%)	807 (49.88%)	754 (46.57%)	776 (47.90%)	0.047
Drinking (n, %)	2622 (40.49%)	801 (49.44%)	606 (37.48%)	618 (38.17%)	597 (36.85%)	<0.001
WC (cm)	84.23 ± 9.90	76.87 ± 8.11	82.42 ± 8.90	86.84 ± 8.59	90.79 ± 8.16	<0.001
BMI (kg/m2)	23.35 ± 3.91	21.22 ± 4.65	22.65 ± 3.18	24.08 ± 3.22	25.44 ± 3.02	<0.001
VAI	1.64 ± 1.67	0.50 ± 0.13	0.91 ± 0.12	1.49 ± 0.23	3.64 ± 2.29	<0.001
SBP (mmHg)	146.34 ± 17.90	147.10 ± 18.07	146.87 ± 18.56	146.12 ± 17.43	145.25 ± 17.45	0.014
DBP (mmHg)	90.25 ± 11.04	87.95 ± 10.86	89.17 ± 11.07	90.63 ± 10.84	93.26 ± 10.69	<0.001
FPG (mmol/L)	90.25 ± 11.04	5.77 ± 1.11	5.88 ± 1.09	6.05 ± 1.29	6.55 ± 2.10	<0.001
TC (mmol/L)	4.96 ± 1.04	4.86 ± 0.97	4.88 ± 1.01	5.04 ± 1.06	5.07 ± 1.12	<0.001
TG (mmol/L)	1.66 ± 1.25	0.78 ± 0.20	1.13 ± 0.24	1.61 ± 0.37	3.14 ± 1.67	<0.001
HDL-C (mmol/L)	1.53 ± 0.44	1.96 ± 0.44	1.59 ± 0.32	1.41 ± 0.28	1.19 ± 0.26	<0.001
LDL-C (mmol/L)	2.86 ± 0.77	2.53 ± 0.68	2.79 ± 0.70	3.04 ± 0.77	3.09 ± 0.78	<0.001
HCY (μmol/L)	20.51 ± 13.65	19.47 ± 11.43	21.02 ± 13.56	20.88 ± 14.71	20.67 ± 14.61	0.004
eGFR (ml/min/1.73m2)	85.91 ± 20.42	86.27 ± 19.42	83.52 ± 21.23	85.93 ± 20.23	87.93 ± 20.52	<0.001
SUA (mmol/L)	7.82 ± 2.00	7.31 ± 1.89	7.61 ± 1.88	7.91 ± 1.91	8.47 ± 2.12	<0.001
Hyperuricemia (n, %)	3983 (61.49%)	808 (49.88%)	935 (57.75%)	1044 (64.48%)	1196 (73.83%)	<0.001
Diabetes (n, %)	1007 (15.54%)	142 (8.77%)	199 (12.29%)	262 (16.18%)	404 (24.94%)	<0.001
CKD (n, %)	358 (5.53%)	77 (4.75%)	90 (5.56%)	102 (6.30%)	89 (5.49%)	0.293
Stroke (n, %)	477 (7.36%)	88 (5.43%)	138 (8.52%)	137 (8.46%)	114 (7.04%)	0.002
Antihypertensive drugs (n, %)	4131 (63.78%)	1017 (62.78%)	1018 (62.92%)	1055 (65.16%)	1041 (64.26%)	0.435
Glucose-lowering drugs (n, %)	256 (3.95%)	31 (1.91%)	51 (3.15%)	69 (4.26%)	105 (6.48%)	<0.001

VAIm visceral adiposity index; BMIm body mass index; WCm circumference; SUAm serum uric acid; SBPm systolic blood pressure; DBPm diastolic blood pressure; FBGm fasting blood glucose; TCm total cholesterol; TGm triglyceride; LDL-Cm low density lipoprotein cholesterol; HDL-Cm high density lipoprotein cholesterol; HCYm homocysteine; eGFRm estimated glomerular filtration rate; CKDm chronic kidney disease.

**Table 2:** Baseline characteristics of study participants across visceral adiposity index (VAI) quartiles in females.

	Total participants	Quartile 1 (< 1.3)	Quartile 2 (1.3-2.0)	Quartile 3 (2.0-3.1)	Quartile 4 (≥ 3.1)	*P-*value
Characteristics	(n = 7238)	n = 1810	n = 1809	n = 1809	n = 1810	
Age (years)	63.78 ± 8.99	65.08 ± 9.35	64.04 ± 9.07	63.54 ± 8.77	62.48 ± 8.58	<0.001
Smoking (%)	399 (5.51%)	94 (5.19%)	105 (5.81%)	93 (5.14%)	107 (5.91%)	0.638
Drinking (%)	383 (5.29%)	124 (6.85%)	104 (5.75%)	83 (4.59%)	72 (3.98%)	<0.001
WC (cm)	83.30 ± 9.79	77.65 ± 9.35	82.60 ± 9.42	85.56 ± 8.95	87.38 ± 8.56	<0.001
BMI (kg/m^2^)	23.77 ± 3.59	22.20 ± 3.63	23.58 ± 3.57	24.40 ± 3.38	24.90 ± 3.15	<0.001
VAI	2.63 ± 2.47	0.93 ± 0.23	1.61 ± 0.20	2.46 ± 0.32	5.53 ± 3.45	<0.001
SBP (mmHg)	150.49 ± 17.52	151.19 ± 17.78	150.42 ± 17.29	150.27 ± 17.33	150.08 ± 17.68	0.235
DBP (mmHg)	87.95 ± 10.38	87.41 ± 10.78	87.63 ± 10.44	87.92 ± 10.06	88.86 ± 10.17	<0.001
FPG (mmol/L)	6.26 ± 1.67	5.97 ± 1.26	6.18 ± 1.66	6.28 ± 1.58	6.62 ± 2.02	<0.001
TC (mmol/L)	5.37 ± 1.11	5.33 ± 1.03	5.41 ± 1.10	5.43 ± 1.13	5.32 ± 1.18	0.003
TG (mmol/L)	1.92 ± 1.23	0.98 ± 0.25	1.41 ± 0.29	1.89 ± 0.40	3.40 ± 1.57	<0.001
HDL-C (mmol/L)	1.61 ± 0.41	1.99 ± 0.40	1.67 ± 0.31	1.50 ± 0.28	1.27 ± 0.27	<0.001
LDL-C (mmol/L)	3.12 ± 0.81	2.89 ± 0.77	3.17 ± 0.80	3.25 ± 0.81	3.17 ± 0.81	<0.001
HCY (umol/L)	15.68 ± 7.28	15.50 ± 7.00	7.75 ± 1.90	15.88 ± 7.96	15.68 ± 7.37	0.483
eGFR (ml/min/1.73m^2^)	90.49 ± 19.74	92.04 ± 18.90	90.35 ± 19.81	90.17 ± 19.55	89.42 ± 20.59	<0.001
SUA (mg/dL)	6.34 ± 1.78	5.79 ± 1.62	6.17 ± 1.66	6.50 ± 1.80	6.90 ± 1.85	<0.001
Hyperuricemia	3713 (51.30%)	668 (36.91%)	879 (48.59%)	1005 (55.56%)	1161 (64.14%)	<0.001
Diabetes	1429 (19.74%)	229 (12.65%)	304 (16.80%)	375 (20.73%)	521 (28.78%)	<0.001
CKD	310 (4.28%)	70 (3.87%)	75 (4.15%)	77 (4.26%)	88 (4.86%)	0.507
Stroke	342 (4.73%)	77 (4.25%)	81 (4.48%)	93 (5.14%)	91 (5.03%)	0.532
Antihypertensive drugs	4650 (64.26%)	1104 (60.99%)	1166 (64.49%)	1166 (64.46%)	1214 (67.11%)	0.002
Glucose-lowering drugs	405 (5.60%)	60 (3.31%)	89 (4.92%)	101 (5.58%)	155 (8.56%)	<0.001

WC, waist circumference; BMI, body mass index; SBP, systolic blood pressure; DBP, diastolic blood pressure; FPG, fasting plasma glucose; TC, total cholesterol; TG, triglycerides;

LDL- C, low-density cholesterol lipoprotein; HDL-C, high-density lipoprotein cholesterol; HCY, homocysteine; SUA, serum uric acid; VAI, visceral adiposity index.

### The association between VAI and hyperuricemia

**Figure 1:**
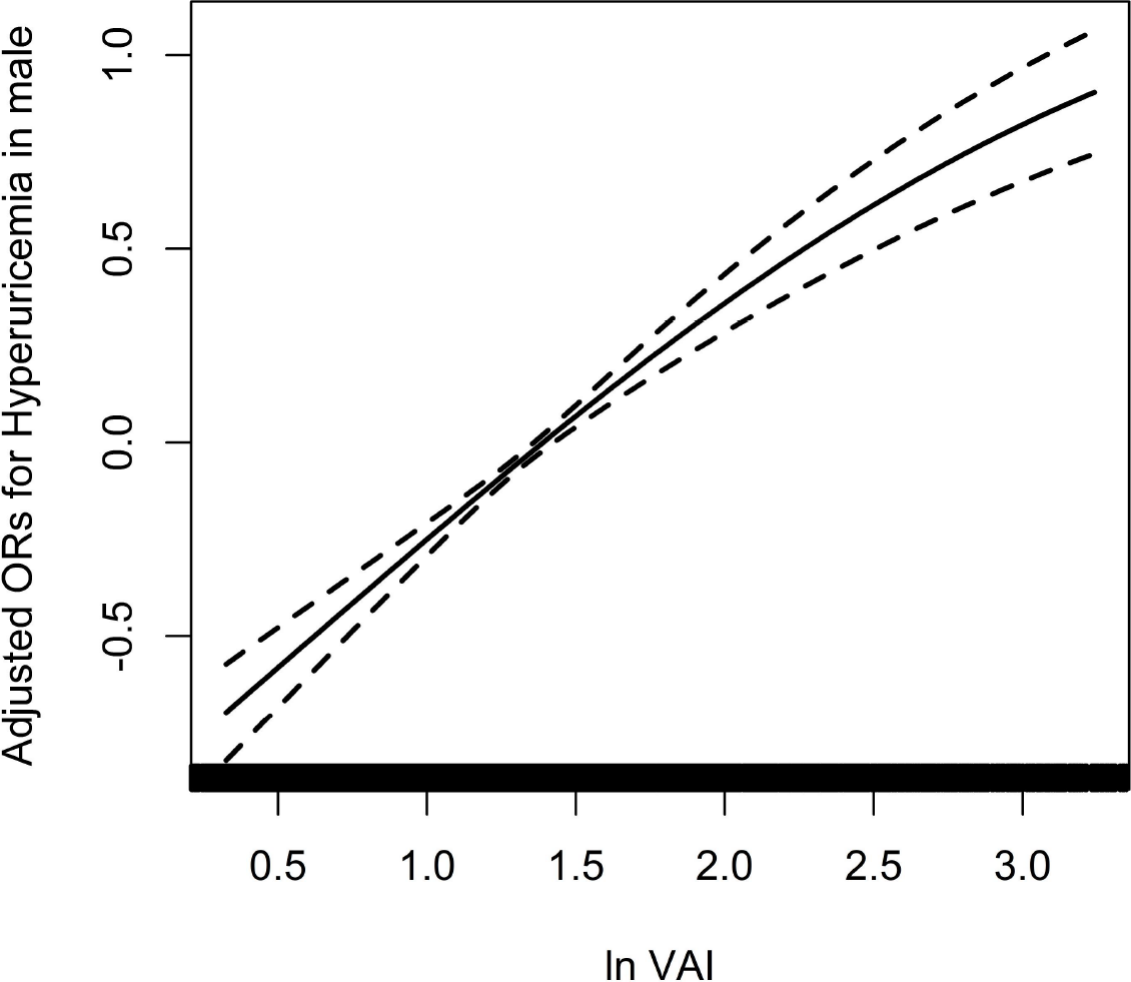
Association between ln VAI and hyperuricemia in males adjusted for age, smoking, drinking, BMI, SBP, DBP, TC, LDL-C, Hcy, eGFR, anti-hypertensive drugs, glucose-lowering drugs.

**Figure 2:**
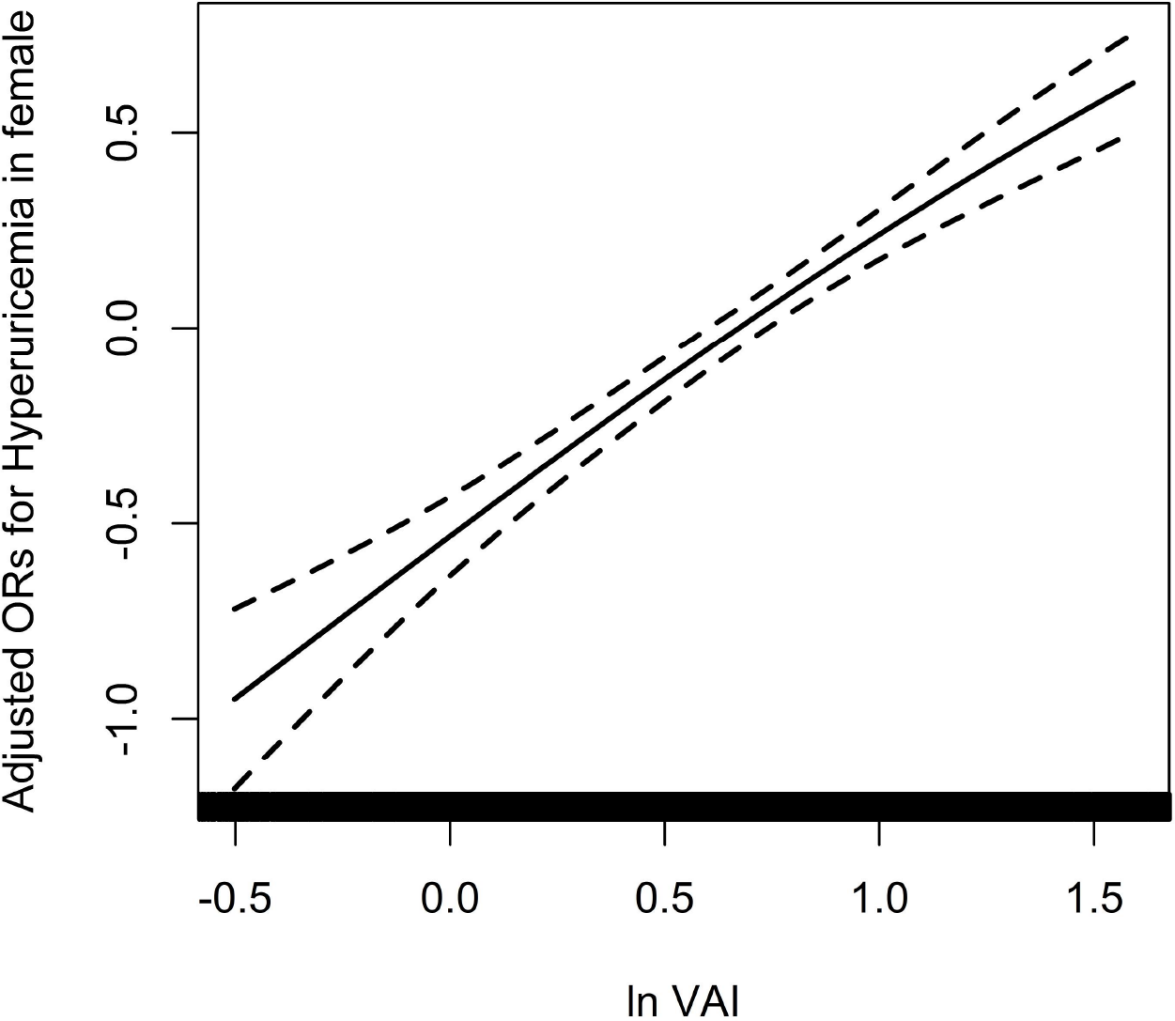
Association between ln VAI and hyperuricemia in females adjusted for age, smoking, drinking, BMI, SBP, DBP, TC, LDL-C, Hcy, eGFR, anti-hypertensive drugs, glucose-lowering drugs.

Because of the non normal distribution of VAI, it was transformed by logarithmic function. **Table 3** displayed the association between ln VAI and hyperuricemia. For males, the risk of hyperuricemia increased by 70% (95% CI: 1.59, 1.83) for each unit of ln VAI in the unadjusted model. In partially and fully adjusted models, ln VAI was independently associated with hyperuricemia, with adjusted ORs of 1.77 (95% CI: 1.65, 1.91) and 1.77 (95% CI: 1.62, 1.94), respectively (*P* for trend <0.001). When ln VAI was converted from a continuous variable to a categorical variable, the relative risk of hyperuricemia in the second, the third, the fourth quartiles were 1.34 (95% CI:1.14, 1.57), 1.82 (95% CI: 1.54, 2.14) and 2.97 (95% CI: 2.48, 3.57) compared to first quartile. For females, the risk of hyperuricemia in the unadjusted model, partially adjusted model and fully adjusted model increased by 1.89times, 1.98 times and 1.88 times, respectively. The relative risk of hyperuricemia in the second, the third, the fourth quartiles were 1.48 (95% CI:1.28, 1.72), 1.99 (95% CI: 1.71, 2.32) and 2.92 (95%CI: 2.50, 3.42) compared to those in first quartile (*P* for trend <0.001). **Figures 1**, **2** showed the association between ln VAI and hyperuricemia clearly using smooth curves in males and females. In addition, we also found that WC and BMI were positively associated with hyperuricemia in hypertensive patients regardless of men and women in the logistic regression model between WC and BMI with hyperuricemia, and the prevalence of hyperuricemia increased with increasing WC and BMI (**Supplementary Tables 1, 2**). Subsequently, we found that the area under curve(AUC) of these three obesity measures for the presence of hyperuricemia were 0.615, 0.588, 0.576 in men (**Supplementary Figure 2**) and 0.620, 0.606, 0.588 in female (**Supplementary Figure 3**), it shows that the correlation between VAI and hyperuricemia is strongest in both female and male.

**Table 3:** Odds ratios and 95% CIs for hyperuricemia according to VAI as continuous variables and quartiles.

	Model 1		Model 2		Model 3	
	OR (95% CI)	*P*	OR (95% CI)	*P*	OR (95% CI)	*P*
**Males**						
Continuous of ln VAI	1.70 (1.59, 1.83)	<0.001	1.77 (1.65, 1.91)	<0.001	1.77 (1.62, 1.94)	<0.001
Quartile of VAI						
Q1(< 0.7)	Ref.		Ref.		Ref.	
Q2(0.7-1.1)	1.37 (1.20, 1.58)	<0.001	1.39 (1.21, 1.60)	<0.001	1.34 (1.14, 1.57)	<0.001
Q3(1.1-2.0)	1.82 (1.58, 2.10)	<0.001	1.90 (1.64, 2.19)	<0.001	1.82 (1.54, 2.14)	<0.001
Q4(≥ 2.0)	2.83 (2.45, 3.29)	<0.001	3.05 (2.61, 3.56)	<0.001	2.97 (2.48, 3.57)	0.001
*P* for trend	<0.001		<0.001		<0.001	
**Females**						
Continuous of ln VAI	1.89 (1.76, 2.04)	<0.001	1.98 (1.84, 2.13)	<0.001	1.88 (1.73, 2.04)	<0.001
Quartile of VAI						
Q1(< 01.3)	Ref.		Ref.		Ref.	
Q2(1.3-2.0)	1.62 (1.41, 1.85)	<0.001	1.67 (1.46, 1.91)	<0.001	1.48 (1.28, 1.72)	<0.001
Q3(2.0-3.1)	2.14 (1.87, 2.44)	<0.001	2.25 (1.96, 2.57)	<0.001	1.99 (1.71, 2.32)	<0.001
Q4(≥ 3.1)	3.06 (2.67, 3.50)	<0.001	3.32 (2.89, 3.81)	<0.001	2.92 (2.50, 3.42)	<0.001
*P* for trend	<0.001		<0.001		<0.001	

Model 1:Adjusted for none.

Model 2:Adjusted for age.

Model 3:Adjusted for age, smoking, drinking, SBP, DBP, FPG, TC, LDL-C, HCY, eGFR, diabetes, stroke, antihypertensive drugs, glucose-lowering drugs.

### Subgroup analysis

**Figure 3:**
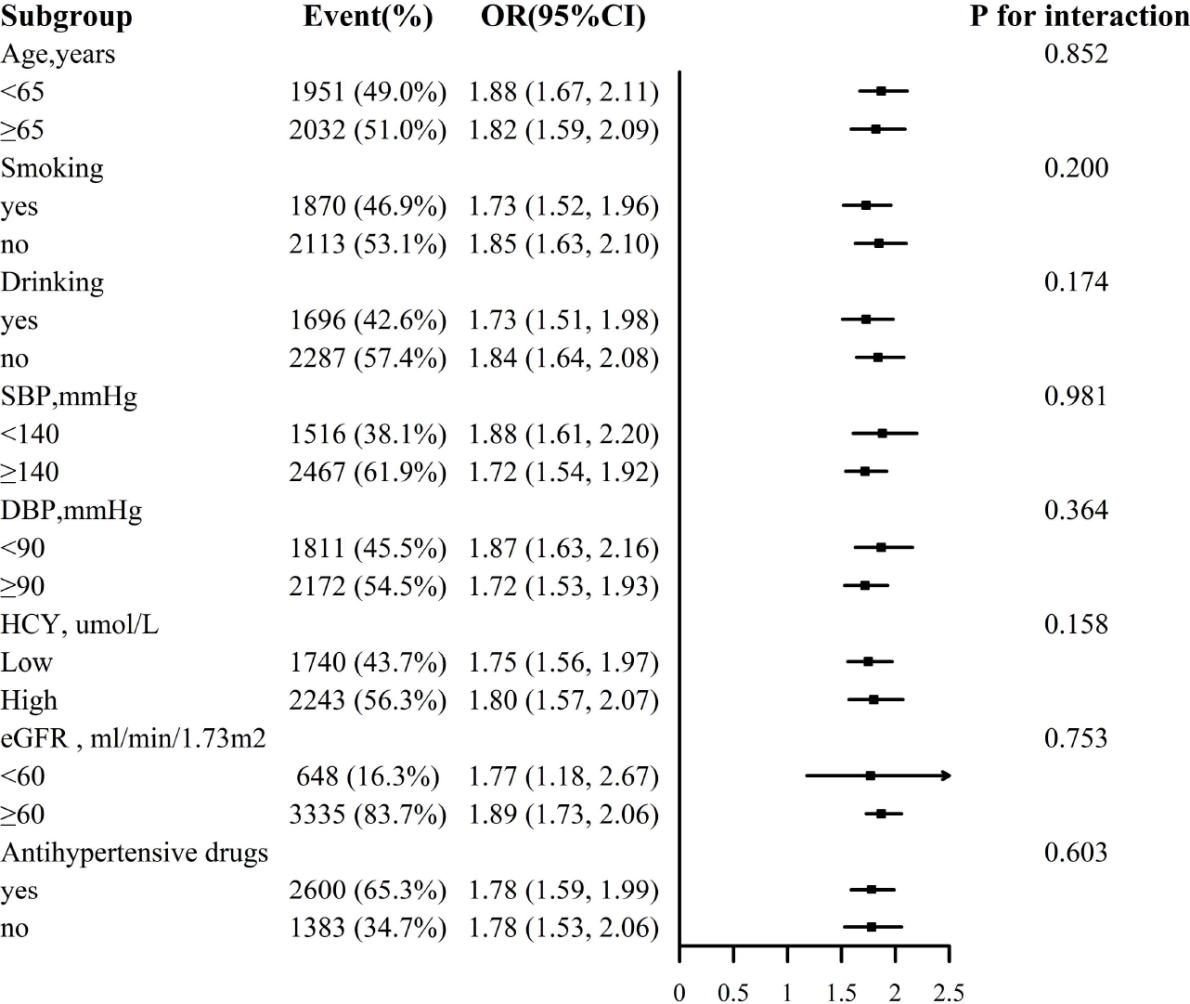
Association between ln VAI and hyperuricemia in males in various subgroups. The models adjusted for age, smoking, drinking, BMI, SBP, DBP, TC, LDL-C, HDL, HCY, eGFR, anti-hypertensive drugs, glucose-lowering drugs except for the stratify.

**Figure 4:**
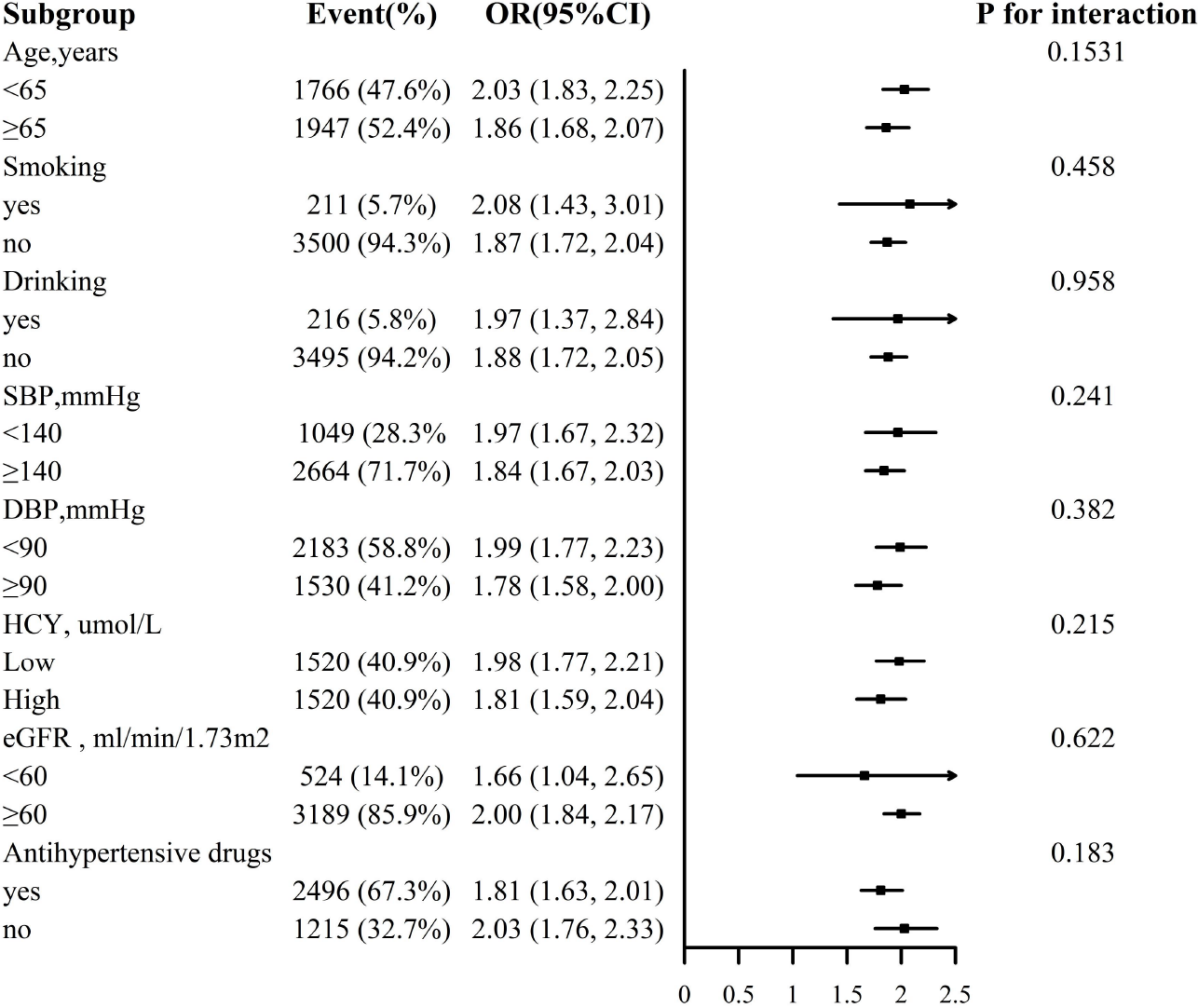
Association between ln VAI and hyperuricemia in females in various subgroups. The models adjusted for age, smoking, drinking, BMI, SBP, DBP, TC, LDL-C, HDL, HCY, eGFR, anti-hypertensive drugs, glucose-lowering drugs except for the stratify.

For the stability of the results, subgroup analysis **Figures 3**, **4** were conducted. Selected subgroups were as follows: age (<65, ≥ 65 years), smoking (no, yes), drinking (no, yes), SBP (<140, ≥ 140mmHg), DBP (<90, ≥ 90 mmHg), Hcy (low, high), eGFR (<60, ≥ 60 ml/min/1.73m2) and antihypertensive drugs (no, yes) (all *P* for interaction > 0.05).

## Discussion

In this large-scale cross-sectional study, we found that VAI was positively associated with hyperuricemia in hypertensive patients. The prevalence of hyperuricemia increased with the increase of VAI. This result might provide a strategy for individuals to reduce uric acid.

According to an epidemiological study in the coastal areas of southeast China, the prevalence of hyperuricemia was 32.4% (25), meanwhile the prevalence of hyperuricemia in the current study was more than 50% in both men and women, which indicating the increasing trend of hyperuricemia.

Previous studies had reported that some obesity traditional indices, such as BMI, WC and waist to height ratio, were associated with hyperuricemia (12, 14, 26). Even earlier, studies had reported the effect of visceral fat accumulation on hyperuricemia and demonstrated that visceral fat was a stronger factor than BMI (27). In recent years, study began to focus on the association between adiposity indices and hyperuricemia. A cross-sectional research involving 633 individuals of health check-up demonstrated that VAI was a better indicator of hyperuricemia than waist circumference. The area under receiver operating characteristic curve of VAI and WC were 0.618 and 0.556, respectively (28, 29). Huimin Dong et al. recruited 7632 adult subjects from the China Health and Nutrition Survey 2009 to investigate whether VAI was independent of hyperuricemia. It turned out that VAI was significantly associated with hyperuricemia, independent of metabolic health and obesity phenotypes (20). In addition, VAI was reported to modify the relationship between urinary albumin/creatinine ratio and serum uric acid. In this study of patients with hypertensive but normal renal function, the risk of hyperuricemia increased with the increase of VAI. However, not all research results were consistent. A study of 174698 adults analyzed the relationship between multiple nontraditional adiposity indices and hyperuricemia, and found a negative correlation between VAI and hyperuricemia (30). The reasons for the discrepancy in results may be attributed to the study population, sample size, statistical analysis, and adjustment factors.

In the current study of hypertensive patients, we found that VAI, an obesity index, was associated with hyperuricemia. The possible mechanisms of hyperuricemia were as follows. First of all, as a hypertensive population, renal microvascular damage and renal ischemia caused by hypertension itself can lead to decreased uric acid excretion and increased production (9). Then, the possible mechanisms of increased risk of hyperuricemia caused by VAI include the following aspects. (1) poor dietary habits and changes in dietary components can lead to obesity. As shown in **Table 1**, patients with high VAI had higher level of BMI and fasting glucose, and higher prevalence of dyslipidemia and hyperuricemia. This suggested that the increase of visceral fat was often accompanied by the metabolic disorder of blood glucose, blood lipid and uric acid. Second, obesity patients tended to be excessive of energy intake, resulting in increased purine synthesis and uric acid production. (2) Fatty acid metabolites could inhibit the excretion of uric acid and promote the increase of serum uric acid level indirectly. (3) Obesity was often associated with insulin resistance, which acted on the kidney and increased uric acid reabsorption and decrease excretion of uric acid, leading to hyperuricemia finally (31, 32). (4) Some obesity-related adipocytokines, such as adiponectin and leptin, have been reported to be associated with hyperuricemia (33, 34). However, the specific physiological mechanism still needs to be confirmed.

Our highlight was the first study to explore the relationship between VAI and hyperuricemia in Chinese hypertensive patients. In addition, large sample size, accurate statistical methods and strict adjustment of confusion factors ensured the reliability of the results. Certainly, some limitations of this study could not be ignored. First, it was not cautious to established causal inference because of the cross-sectional nature of our study and further prospective studies are needed to explain the exact role of visceral fat accumulation in metabolism of serum uric acid and progression of hyperuricemia. Secondly, present study only evaluated the relationship between VAI and hyperuricemia in Chinese hypertensive patients, whose results might not be extrapolated to other populations. Finally, we did not consider the effect of diet and drugs on serum uric acid level due to the limitation of data.

To sum up, this study demonstrated that VAI was closely related to hyperuricemia in hypertensive patients, which might provide a strategy for reducing uric acid in for obesity patients in clinical practices.

## Data availability statement

The datasets presented in this article are not readily available because data access is obtained according to individual contributions to the study. Requests to access the datasets should be directed to the corresponding authors. Requests to access these datasets should be directed to XC, xiaoshumenfan126@163.com.

## Ethics statement

The study was conducted in accordance with the Declaration of Helsinki and was approved by the Ethics Committee of the Anhui Medical University Biomedical Institute. The patients/participants provided their written informed consent to participate in this study.

## Author contributions

All authors contributed to: (1) substantial contributions to conception and design, or acquisition of data, or analysis and interpretation of data, (2) drafting the article or revising it critically for important intellectual content, and, (3) final approval of the version to be published. All authors contributed to the article and approved the submitted version.

## Supplementary material

Supplementary Figure 1 Flow charts of participants.

Supplementary Figure 2 ROC curves for incident hyperuricemia comparing VAI, BMI, and WC in female.

Supplementary Figure 3 ROC curves for incident hyperuricemia comparing VAI, BMI, and WC in male.

Supplementary Table 1 Odds ratios and 95% CIs for hyperuricemia according to WC as continuous variables and quartiles

Supplementary Table 2 Odds ratios and 95% CIs for hyperuricemia according to BMI as continuous variables and quartiles.
