# Corrigendum: The elevated visceral adiposity index increases the risk of hyperuricemia in Chinese hypertensive patients: a cross-sectional study

**DOI:** 10.3389/fendo.2023.1268274

**Published:** 2023-10-17

**Authors:** XiaoLi Song, Hui Liu, Jian Zhu, Wei Zhou, Tao Wang, Chao Yu, Lingjuan Zhu, Xiaoshu Cheng, Huihui Bao

**Affiliations:** ^1^ Department of Cardiovascular Medicine, The Second Affifiliated Hospital of Nanchang University, Nanchang, Jiangxi, China; ^2^ Central Hospital of Huanggang, Huanggang, Hubei, China; ^3^ Qiu Kou Town Central Health Center, Wuyuan, Jiangxi, China; ^4^ Jiangxi Provincial Cardiovascular Disease Clinical Medical Research Center, Nanchang, Jiangxi, China; ^5^ Center for Prevention and Treatment of Cardiovascular Diseases, The Second Affifiliated Hospital of Nanchang University, Nanchang, Jiangxi, China

**Keywords:** visceral adiposity index, uric acid, hyperuricemia, hypertension, obesity

## Incorrect affiliation

In the published article, there was an error in affiliation(s) [1,2]. Instead of “[^1^Central Hospital of Huanggang, Huanggang, Hubei, Nanchang, China,^2^Department of Cardiovascular Medicine, The Second Affifiliated Hospital of Nanchang University, Nanchang, Jiangxi, China]”, it should be “[^1^Department of Cardiovascular Medicine, The Second Affifiliated Hospital of Nanchang University, Nanchang, Jiangxi, China, ^2^Central Hospital of Huanggang, Huanggang, Hubei, China]”.

The authors apologize for this error and state that this does not change the scientific conclusions of the article in any way. The original article has been updated.

## Error in Author List

**Please also check that the initials used in the Author Contributions section or elsewhere in the article are correct.**

In the published article, there was an error in the author list, and author [XiaoLi Song, Hui Liu] was erroneously [[arranged]. The corrected author list appears below.

[XiaoLi Song ^1#^, Hui Liu ^2#^, Jian Zhu ^4^, Wei Zhou ^3,5^, Tao Wang ^3,5^, Chao Yu ^3,5^, Lingjuan Zhu ^3,5^, Xiaoshu Cheng ^1,3,5^ and Huihui Bao ^1,3,5,*^].

The authors apologize for this error and state that this does not change the scientific conclusions of the article in any way. The original article has been updated.

