# Supplementary material for: The elevated visceral adiposity index increases the risk of hyperuricemia in Chinese hypertensive patients: A cross-sectional study
Source: Front Endocrinol (Lausanne). 2022 Dec 15;13:1038971. doi: 10.3389/fendo.2022.1038971 (PMC9798281; doi:10.3389/fendo.2022.1038971)
Supplement: Supplementary Figure 1 — Flow charts of participants. [file DataSheet_1.pdf]

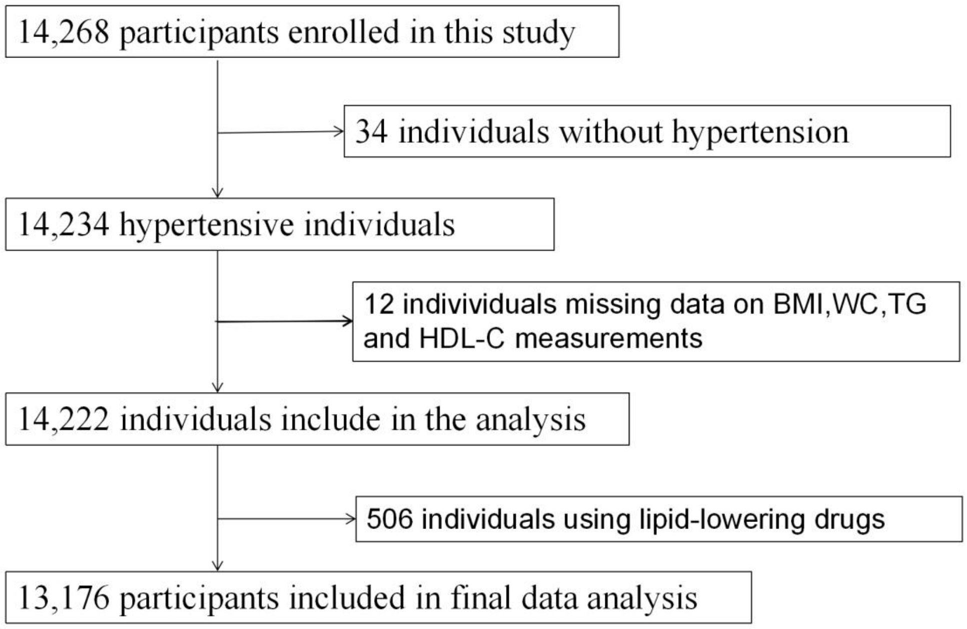

Supplementary Figure 1. Flow chart of participants

Figure S2.ROC curves for incident hyperuricemia comparing VAI, BMI, and WC in female.

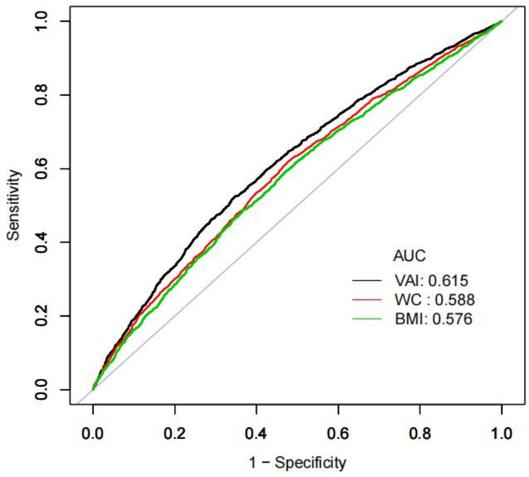

Figure S3. ROC curves for incident hyperuricemia comparing VAI, BMI, and WC in male.

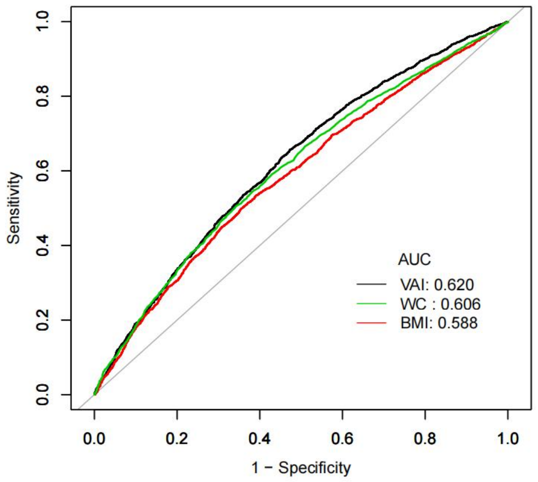

Table S1.Odds ratios and 95% CIs for hyperuricemia according to WC as continuous variables and quartiles.

	Model 1		Model 2		Model 3	
	OR (95% CI)	*P*	OR (95% CI)	*P*	OR (95% CI)	*P*
**Males**						
Continuous of WC	1.03 (1.03, 1.04)	<0.001	1.03 (1.03, 1.04)	<0.001	1.03 (1.03, 1.04)	<0.001
Quartile of WC						
Q1(<79.9)	Ref.		Ref.		Ref.	
Q2(80.0-88.5)	1.43 (1.27, 1.62)	<0.001	1.46 (1.29, 1.65)	<0.001	1.47 (1.28, 1.69)	<0.001
Q3(≥88.6)	1.95 (1.72, 2.20)	<0.001	2.00 (1.76, 2.28)	<0.001	1.94 (1.67, 2.26)	<0.001
P for trend	<0.001		<0.001		<0.001	
**Females**						
Continuous of WC	1.04 (1.03, 1.04)	<0.001	1.04 (1.04, 1.05)	<0.001	1.04 (1.04, 1.05)	<0.001
Quartile of WC						
Q1(<79.0)	Ref.		Ref.		Ref.	
Q2(79.1-87.4)	1.51 (1.35, 1.69)	<0.001	1.59 (1.41, 1.78)	<0.001	1.63 (1.43, 1.85)	<0.001
Q3(≥87.5)	2.38 (2.12, 2.67)	<0.001	2.54 (2.26, 2.86)	<0.001	2.54 (2.23, 2.91)	<0.001
P for trend	<0.001		<0.001		<0.001	

Model 1:Adjusted for none.

Model 2:Adjusted for age

Model 3:Adjusted for age, smoking, drinking, SBP, DBP, FPG, TC, LDL-C, HCY, eGFR, diabetes, stroke, antihypertensive drugs, glucose-lowering drugs

Table S2.Odds ratios and 95% CIs for hyperuricemia according to BMI as continuous variables and quartiles.

	Model 1		Model 2		Model 3	
	OR (95% CI)	*P*	OR (95% CI)	*P*	OR (95% CI)	*P*
**Males**						
Continuous of BMI	1.07 (1.06, 1.09)	<0.001	1.08 (1.06, 1.10)	<0.001	1.07 (1.05, 1.09)	<0.001
Quartile of BMI						
Q1(<21.6)	Ref.		Ref.		Ref.	
Q2(21.6-24.7)	1.37 (1.22, 1.55)	<0.001	1.41 (1.24, 1.59)	<0.001	1.39 (1.21, 1.60)	<0.001
Q3(≥24.7)	1.84 (1.62, 2.08)	<0.001	1.93 (1.69, 2.21)	<0.001	1.83 (1.57, 2.14)	<0.001
P for trend	<0.001		<0.001		<0.001	
**Females**						
Continuous of BMI	1.09 (1.07, 1.10)	<0.001	1.11 (1.09, 1.13)	<0.001	1.11 (1.09, 1.13)	<0.001
Quartile of BMI						
Q1(<22.1)	Ref.		Ref.		Ref.	
Q2(22.1-25.1)	1.31 (1.17, 1.47)	<0.001	1.47 (1.31, 1.65)	<0.001	1.44 (1.26, 1.63)	<0.001
Q3(≥25.1)	2.02 (1.80, 2.27)	<0.001	2.38 (2.11, 2.69)	<0.001	2.36 (2.06, 2.70)	<0.001
P for trend	<0.001		<0.001		<0.001	

Model 1:Adjusted for none.

Model 2:Adjusted for age

Model 3:Adjusted for age, smoking, drinking, SBP, DBP, FPG, TC, LDL-C, HCY, eGFR, diabetes, stroke, antihypertensive drugs, glucose-lowering drugs
